# Toxicology as a diagnostic tool to identify the misuse of drugs in the perinatal period

**DOI:** 10.3389/fped.2022.1071564

**Published:** 2023-02-08

**Authors:** Joseph Jones

**Affiliations:** United States Drug Testing Laboratories, Des Plaines, IL, United States

**Keywords:** newborn toxicology, maternal substance use, substance abuse, prenatal drug exposure, umbilical cord testing, meconium testing, forensic testing

## Abstract

The use, misuse, and abuse of substances are a continued public health concern in this country and around the world. Perinatal exposure to substances of abuse is associated with several long-term negative consequences for the neonate. Limited resources exist to assist perinatal health professionals on this very complex subject. The purpose of this document is to provide additional information about selecting monitoring protocols, the specifics of appropriate testing methodologies, and the interpretation of toxicological findings. Understanding these concepts better allows perinatal healthcare professionals to be a voice for the voiceless in order to protect and enrich lives during this unprecedented opioid epidemic.

## 1. Introduction

The use, misuse, and abuse of substances, including both prescription opioids and non-prescription opioids, are a continued public health concern (1). Prenatal exposure to these substances may lead to a number of negative health consequences, including neonatal opioid withdrawal syndrome (NOWS), a subcategory of neonatal abstinence syndrome (NAS); premature birth; stillbirth; and an array of other long term negative health consequences (2, 3). Additionally, children of parents suffering from substance use disorders are at a three-fold higher risk of experiencing child maltreatment (4, 5).

A long-standing objective of the HealthyPeople initiative has been to promote an increase of maternal abstinence from illicit substances. HealthyPeople 2030 (6) has targeted an increase from the baseline of 93% of pregnant women reporting abstinence in the National Survey on Drug Use and Health (NSDUH) to 95.3% reporting abstinence by 2030. The most recent findings published in the aggregated 2018–2019 NSDUH was 94.0% [95% CI: 92.9%, 95.6%] (CBHSQ, 2020). This improvement was not statistically significant but it is in the desired direction.

The 2020 NSDUH reported 8.3% (SE 2.05) of pregnant mothers claimed to have used an illicit substance in the past month, which was up from 5.8% (SE 1.04) in 2019 (7). A good portion of these mothers are at or near the poverty level and without private insurance. This highlights the fact that this population is very vulnerable with regard to inadequate access to prenatal care and treatment for substance use disorders and presents an opportunity for public health intervention efforts.

Fulfilling the objectives of HealthyPeople 2030 suggests that perinatal healthcare professionals must understand the scope and extent of prenatal substance exposure (8). Specifically needed are processes to provide effective prevention efforts, identify exposure in both an epidemiological and specific case perspective, recognize medical issues associated with perinatal exposure to substances, provide protection for the infant, and refer the exposed infants for appropriate follow-up when needed (8). To accomplish these objectives, the perinatology professional must obtain credible information. Questionnaires and the analysis of various biological specimen types are currently the two approaches for obtaining perinatal substance exposure information. Using questionnaires to obtain credible perinatal substance exposure information is very difficult due to the potential promotion of stigma and guilt which undermines a patient/healthcare professional trust relationship and the potential legal ramifications. Testing biological specimens have less than perfect sensitivity due primarily to detection window limitations.

Further complicating testing biological specimens is the complexity of the maternal-fetal dyad. The placenta, a temporary organ, resides between the mother and baby serving as an interface. Molecules, including substances of abuse and their metabolites, are transported through the placental barrier through simple diffusion (such as oxygen and carbon dioxide) and more complex transport mechanisms (9). The placenta is also a structure that is capable of metabolizing certain compounds that in some cases varies with gestational age (9). The structure of the human placenta is sufficiently different from other mammals which limits generalizability of the study of transport functions in animal models (9). Random controlled trials of prenatal exposure to substances of abuse are lacking due to the ethical considerations of providing pregnant persons a known toxic compound for research purposes.

Testing of biological specimens to monitor perinatal substance exposure is a very specialized field. Limited resources are available to perinatal health professionals to design perinatal substance exposure-monitoring strategies and assist with interpretation. The author consults routinely in cases where Child Protection Service action was taken based on specimens analyzed without chain of custody, presumptive positive results that have not been confirmed by a sufficiently specific method, and lacking review of the medical record to determine if the positive was due to hospital administered medicine. The aim of this manuscript is three-fold. We will review both commonly available options for perinatal substance monitoring and important concepts to consider when designing a monitoring policy, as well as discuss some frequently asked questions regarding the interpretation of newborn toxicology results.

## 2. Detection and monitoring of perinatal substance exposure

### 2.1. Questionnaire

The American College of Obstetricians and Gynecologists (ACOG) recommends screening all pregnant women for substance use with a validated questionnaire for the purpose of intervention and referral (10). There are several validated questionnaires available for use, such as the Drug Abuse Screening Test (DAST-10), the 4P's, Substance Use Risk Profile-Pregnancy, the CRAFFT screening tool, NIDA Quick Screen, and the Wayne Indirect Drug Use Screener (10). Strengths associated with the use of these tools are that they are inexpensive, quick to administer, and can monitor for substance use throughout the entire perinatal period (8, 10). However, limitations include recall bias and under-reporting due to stigma and fear of legal repercussions (8–12). Many unvalidated “local questionnaires” are in use, which may unwittingly negatively impact sensitivity and specificity (13).

### 2.2. Biological specimen types

#### 2.2.1. Maternal urine

Maternal urine testing is the primary biological specimen type used for monitoring maternal substance use during the perinatal period, including at intake upon arrival at the birthing center (14, 15). Perinatal professionals have used urine testing for many decades. Urine testing has proven to be a reliable specimen type, many laboratories are proficient with the testing procedures, and costs are low compared to other specimen types. Additionally, many clinicians have sufficient experience with the interpretation of the results.

Many laboratories test specimens in a clinical environment as opposed to a forensic environment. Presumptive positive specimens are routinely unconfirmed using a definitive technique, processed without documented chain of custody, and destroyed in a few days regardless of the outcome of the test (which eliminates the possibility of a retest when there is a question about the accuracy of a result). Under these circumstances, these tests are satisfactory to utilize for research or as a screening tool to initiate brief intervention, further testing, or additional monitoring. These specimen results, if not performed using forensic protocols (maintaining a documented chain of custody and automatic confirmation of presumptive positive specimens), should not initiate negative action towards the mother and/or child.

#### 2.2.2. Maternal blood

In this environment, maternal blood is typically not a specimen type of choice for drug testing. The collection protocol is invasive and presents an unnecessary biohazard risk to transportation and laboratory staff; the detection windows are very short (shorter than urine); and the analysis is very expensive. There are new tests that show promise in this environment, such as phosphatidylethanol (PEth) in whole blood or dried blood spots. PEth is a direct ethanol biomarker that detects prenatal ethanol exposure and has a detection window measured in weeks rather than days (16).

#### 2.2.3. Maternal hair

Maternal hair is a specimen type that offers a very long detection window. Analytes incorporate into hair by three main routes. First is environmental exposure. In an environment where a drug is used, smoked, handled, manufactured, or prepared, the environment becomes contaminated, and the drug over time will transfer to the hair. Next is consumption. When a user consumes a drug, the sweat and sebum contain drug and drug metabolites, and as these fluids bathe the hair shaft these analytes deposit on the hair. Lastly, also following consumption, the blood, which contains drug and drug metabolites, deposits the analytes into the root. Once in or on the hair, the analytes bind to proteins and pigments in the hair and remain for an extended period.

Hair testing has several advantages. The collection procedure is simple and noninvasive. The collection may directly observe the donor without gender issues (15, 17). The collector may execute the collection outside of clinical settings. The detection window of drug in hair is months instead of days depending on the source of hair and the compound of interest.

There are several limitations of using hair as a specimen type for drug testing. Cosmetic treatment may interfere with analysis depending on the substance or treatment. These processes contain varying amounts of reducing and/or oxidizing agents, which may alter the structure of the compound of interest (18, 19). The analysis requires a complex specimen preparation, which makes the testing expensive (20). Lastly, there is a potential for observing a positive maternal hair test result due to external contamination or environmental exposure. While providing important information concerning the maternal environment, it does not provide specific evidence of prenatal exposure (15).

#### 2.2.4. Newborn urine

For many years, newborn urine was the primary strategy to objectively identify prenatal drug exposure. The advantages of newborn urine testing are similar to the advantages listed for maternal urine testing, but there are several limitations to testing newborn urine.

Several limitations exist regarding newborn urine to monitor prenatal substance exposure (15). The ideal newborn urine specimen is the neonate's first urine void, and it is difficult to know if the specimen captured was indeed the first void. Missing the first urine void is commonplace. The newborn produces a limited volume of urine with the first void. This results in an excess of specimen rejections due to insufficient quantity for testing. Dilute newborn urine is typical, which shortens an already short detection window even further. Collection protocols are clumsy, and the adhesives are irritating to delicate newborn skin. These limitations led to the development of other testing strategies, such as newborn hair, meconium, and umbilical cord tissue segments as alternatives for monitoring prenatal substance exposure.

#### 2.2.5. Newborn hair

Newborn hair testing offers many of the benefits mentioned in the maternal hair discussion above. Hair forms in the third trimester, and substances and their metabolites may become entrapped in the hair, thus offering a long window of detection (15). References to using newborn hair for prenatal drug exposure appears in the literature (21–24), but its use is not routine. While newborn hair provides a long window of detection and is a simple non-invasive collection process, newborn hair is routinely not present or in sufficient quantity to complete all testing. Approximately one-fourth of all children born do not have sufficient hair for testing. This obstacle limits the use of hair as a primary strategy for most routine prenatal substance monitoring programs (20).

#### 2.2.6. Newborn meconium

The first alternative specimen to routinely replace newborn urine as the specimen of choice for newborn toxicology was meconium (14, 25–28). Meconium is the first fecal matter excreted by the newborn and is a complex and highly variable material composed primarily of mucopolysaccharides, water, bile, salts, bile acids, epithelial cells, and other lipids (29). Meconium begins to form near mid-term of the pregnancy with the majority forming after week 38. As the laboratory equipment and laboratory processes evolved to meet the demand and challenges of high throughput workplace drug testing resulting from the Federal Drug Free Workplace Act in the late 1980s, these processes and equipment were available to develop feasible and practical newborn toxicology testing strategies.

The primary advantage of meconium is the long detection window which includes the entire last trimester. Advantages include a non-invasive collection procedure. Additionally, enough specimen is available for testing in most cases and there are several laboratories available to perform the testing. These advantages have, over time, resulted in meconium becoming the gold standard of newborn toxicology (11).

As with any testing protocol, there are several limitations to using meconium. Limitations include the lack of detection of prenatal exposure in early pregnancy. The detection window is bound by the time of the formation of meconium and the fact that most of the meconium production occurs in the last few weeks, thus diluting earlier use (11). The distribution of analytes in meconium is heterogeneous. Therefore, the ideal collection procedure includes all passages of meconium. The transition from meconium to milk stool can be difficult to discern in some cases. The collection procedure is a multi-step process, which requires multiple collections by multiple collectors over multiple shifts and sometimes over multiple days. Meconium collection can be a very timely and expensive process. Lastly, all laboratories do not have the capability to adequately execute testing on this very difficult specimen type. While there are laboratories available for meconium testing, the number of competent laboratories remains limited.

#### 2.2.7. Newborn umbilical cord

Concheiro and Huestis (11) noted that due to the number of limitations to using meconium in an organization's newborn toxicology program, umbilical cord tissue segment testing was developed as an alternative specimen type to meconium. Testing newborn umbilical cord for substances of abuse has been gaining traction in the newborn toxicology environment over the past 15 years. The development of umbilical cord testing was a direct response to an unacceptable number of meconium specimens rejected or canceled due to low sample volume (30, 31).

Umbilical cord as a specimen for newborn toxicology has several advantages (30–34). There is an abundance of specimen available for each birth, making umbilical cord collection truly universal. Table 1 provides a summary of examples in the literature demonstrating the extent of sample volume compliance when using meconium. The specimen collection and transfer to the laboratory occurs immediately following birth, which improves turnaround times. Analytes appear evenly distributed throughout the entire length of the cord. Analytes in meconium appear heterogeneously distributed, requires collection of the entire passage, and requires mechanical mixing (34, 39). Umbilical cord collection is a simple single-step procedure whereas meconium requires multiple collectors making multiple collections over multiple shifts and/or days.

**Table 1:** Examples in the literature that demonstrate the extent of sample volume compliance using meconium for monitoring substances.

Year	Study	Enrolled	Unavailable	% Unavailable
1999	Arendt et al. (35)	218	61	27.9
2001	Lester et al. (14)	8,527	3,284	27.8
2003	Derauf et al. (36)	546	110	20.1
2005	Eylera et al. (37)	51	5	9.8
2010	Gray et al. (38)	102	14	13.7

A disadvantage of using umbilical cord is that the concentrations of detectable substances and their metabolites are low, which requires more expensive laboratory equipment to achieve cutoffs that provide adequate sensitivity (30, 31, 40). This makes the analysis more expensive than meconium, but when controlling for the expense of multiple collections, missed opportunities, and turnaround time, the overall expense of using umbilical cord is comparable with meconium.

## 3. Concepts to consider when designing a perinatal substance monitoring policy

### 3.1. Questionnaire vs. biological specimen

An important question to ask when developing a newborn toxicology policy for your organization is whether to use a questionnaire and/or biological specimen. Several examples exist in the literature that compare the effectiveness of various self-report questionnaires and various validated biological specimen analyses (41–47). Following a review of the existing literature, the authors compared the rates of self-reported prenatal substance exposure and the presence of corresponding biomarkers using a variety of specimen types (13). In each instance in the literature reviewed, self-reported substance exposure was under-reported when compared to biological specimen analysis (13). Behnke et al. (8) noted that no single monitoring method was perfectly sensitive and specific and therefore recommended coupling questionnaire and biological analysis to improve the probability of identifying perinatal substance exposure.

### 3.2. Which biological specimen to choose

Take care when selecting the biological specimen type to use in a perinatal substance exposure program. Detection of a substance in a maternal specimen provides evidence of maternal exposure but does not necessarily provide conclusive evidence of substance exposure to the neonate (11). The detection of a substance or its metabolite in a specimen originating from the neonate provides conclusive evidence of prenatal exposure to a substance. Additionally, the Supreme Court of the United States (48) opined that using a maternal specimen that could result in legal repercussions requires the consent of the mother. This rationale does not extend to the specimens obtained from the neonate. The Keeping Children and Families Safe Act (Public Law 108-36) imposed a requirement to report the detection of prenatal illicit drug exposure to the State. Under these conditions, best practices necessitate that these tests, when ordered due to reasonable suspicion, should satisfy basic forensic tenants such as the maintenance of chain of custody and confirmation of presumptive positive results. Table 2 lists advantages and disadvantages for various specimen types used for monitoring prenatal substance exposure.

**Table 2:** Advantages and disadvantages of various specimen types commonly used for monitoring prenatal substance exposure.

Specimen type	Advantages	Disadvantages
Maternal urine	Test is inexpensive Most understood Analysis is simple and may be performed in house	Short detection window Gender issues at collection Requires maternal consent
Maternal blood		Short detection window Invasive collection Requires maternal consesnt Testing is very expensive Difficult and challenging analysis
Maternal hair	Long detection window Moderate costs Noninvasive collection	Requires maternal consent Cosmetic treatment issues Difficult and challenging analysis
Newborn Urine	Low cost Analysis is simple and may be performed in house	Cumbersome collection Easy to miss first void Very short detection window Insufficient quantity of specimen
Newborn hair	Long detection window Moderate costs	Insufficient quantity of specimen Difficult and challenging analysis
Meconium	Long detection window Moderate costs	Quantity is not sufficient for many babies May passed *in utero* due to fetal stress Requires multiple collections May require days to pass enough specimen for testing Analytes are not distributed evenly Difficult and challenging analysis
Umbilical Cord	Long detection window Moderate costs Plenty of specimen for every baby Specimen is available immediately following birth Collection is a single step procedure Analytes are distributed evenly throughout the length of the cord	Difficult and challenging analysis Requires newer more sensitive laboratory instruments

### 3.3. Analysis of biological specimens

An effective substance exposure monitoring program requires a sensitive and specific testing strategy. While some research and clinical environments rely on a single immunoassay or single mass spectrometric protocol, which is adequate under research and/or clinical conditions. Newborn toxicology cases routinely transition from a clinical situation to a forensic situation (49). Policy makers who design workflows that rely on results generated without using commonly accepted forensic standard protocols (maintenance of chain of custody and confirmation of presumptive positive specimens) to reduce costs are acting in a scientifically irresponsible manner.

### 3.4. Screening or initial testing

Several techniques exist to monitor newborn specimens to preliminarily detect prenatal substance exposure. The most common initial tests utilize the sensitivity, speed, and cost effectiveness of a variety of immunoassay techniques, such as enzyme linked immunosorbent assay (ELISA), enzyme multiplied immunoassay test (EMIT®), cloned enzyme donor immunoassay (CEDIA), Diagnostics Reagents Inc immunoassay (DRI®), or homogenous enzyme immunoassay (HEIA™) (50–52). These methods provide a quick and economical way to identify negative specimens with adequate sensitivity, which in turn allows the laboratory to focus its attention on the presumptive positive specimens.

Several methods exist in the literature and commerce that utilize a mass spectrometric initial test protocol. Most common are liquid chromatography tandem mass spectrometry (LCMSMS) and liquid chromatography time of flight mass spectrometry (LCTOFMS) (11, 39, 53). These techniques allow for a higher degree of specificity over immunoassay at the expense of time and/or cost. However, the laboratory should confirm presumptive positive results obtained from these methods using a second protocol before reporting results to the State.

### 3.5. Confirmation testing

Once the laboratory obtains a presumptive positive test result by an adequate initial test, a confirmation test follows to confirm the presence of the specific analyte identified with the initial test. Currently, mass spectrometric techniques are the gold standard for this purpose due to the technique's high degree of sensitivity and specificity. Confirmation testing should use a second portion of the original specimen, regardless of the method used for initial testing. This is a best practice to rule out frame shift errors.

### 3.6. Importance of confirmation testing

Gray and Huestis (15) said, “Confirmation of positive screening results is essential.” Confirmation testing serves two primary purposes. First, the process of confirming an initial presumptive positive test by analyzing a second aliquot (a portion of a specimen used for analysis) mitigates the possibility of a frame shift error or sample switching in the initial testing process. Following a frame shift error during the initial testing process, the confirmation results will not agree with the initial test, thereby alerting the testing personnel of a potential error. Second, the use of two different analytical methodologies or procedures to arrive at the same result dramatically increases the analytical specificity of the entire process. This concept is even more important when considering that newborn biological tests represent a once in a lifetime opportunity to protect and enrich the life of the neonate. The use of a screen and confirm strategy while maintaining a documented chain of custody ensures the integrity of the identity of the specimen and ensures the accuracy of the result, thereby protecting the maternal-child dyad from erroneous results. These are the cornerstone principles of producing a forensically defensible result.

### 3.7. External oversight

External oversight of laboratory operations is an important best practice in our field, but all external oversight providers are not the same. There are multiple options of external oversight to choose from (such as CLIA, CAP, COLA), and the laboratory may select the oversight provider that best fits its geographic and/or regulatory needs. However, there are a select few options that provide oversight from the context of producing a forensically defensible result [such as CAP-FDT, NYDOH-Forensic Toxicology, ISO17025; (54)].

Clinical laboratories knowingly or unknowingly operating in a forensic environment without appropriate forensic oversight can expose all stakeholders involved to unexpected levels of risk (55). The Ontario Ministry of the Attorney General (55) reported how a well-respected laboratory staffed and managed by a highly competent team from a research and clinical perspective created a situation that required years of litigation and review of thousands of cases over multiple decades. Relevant external oversight would prevent this unfortunate outcome. It is important that newborn toxicology policymakers understand these differences and choose their testing laboratory accordingly.

## 4. Interpretation of biological specimen test results

Following the receipt of a biological specimen test result, you require an interpretation. Is the reported outcome the result expected? Is there a reasonable explanation for the result? Is a reasonable explanation lacking? These, among others, are very important questions that perinatal professionals address routinely.

### 4.1. Does a negative result infer abstinence?

A negative result is not conclusive evidence of abstinence. There are many reasons why a particular outcome is negative, especially considering the complex biology of a maternal-fetal system. The most common scenario in the experience of this authoris the test ordered does not include the specific substance in question. Standardization of newborn toxicology testing is currently lacking and each laboratory performing newborn toxicology testing have unique testing panels and cutoffs. The ordering and/or result interpretation professional should be knowledgeable of the substances included in the test ordered. Additionally, a negative result may be due to the use of the substance beyond the detection window of the specimen type or the donor consumed an insufficient amount of the substance to generate a positive result.

### 4.2. What are the detection windows?

The amount of time represented for a biological specimen result is the detection window or window of detection. Each specimen type has a commonly agreed upon detection window (17, 30, 31, 50). The windows of detection for each specimen type appear previously, and these detection windows appear in Table 3 for convenient comparison.

**Table 3:** Commonly accepted windows of detection by specimen type.

Specimen type	Detection window	Comment
Maternal urine	2–5 days	For most drugs, most used
Maternal blood	1–2 days	Uncommon for this purpose due to short detection window and high expense
Maternal hair	Up to approximately 12 weeks	Using 1.5 inches of hair Hair color and cosmetic treatment are variables May detect environmental exposure
Newborn Urine	1–2 days	First void is best practice Very dilute
Newborn hair	8 weeks	Detection starts when hair starts forming Many babies do not have enough hair
Meconium	Up to approximately 20 weeks	Difficult multi step collection process Issues with sample amount compliance
Umbilical Cord	12 weeks	Developed to mirror meconium Universal specimen type

### 4.3. Is there a relationship between the reported concentration and the amount of substance consumed?

Several variables influence the observed concentration of any analyte in a reservoir specimen type, a specimen type where analytes may accumulate over time such as umbilical cord, meconium, hair, or urine. Currently, the scientific literature does not support using the reported concentrations to predict the amount of substance ingested, time of ingestion, or the frequency of ingestion (11, 56) even under tightly controlled research conditions (57).

### 4.4. Are medications provided to the mother or newborn detectable in newborn specimens?

Perinatal professionals should review the results to determine if the positive result aligns with the medical record. Detection of medications provided to the mother prior to birth may occur in newborn specimen types, including medications provided during labor and delivery (58). Medications given to the neonate postnatally may also appear in specimens collected following birth, such as meconium or newborn urine.

### 4.5. Was chain of custody documented, and was confirmation testing performed?

US physicians must notify state child protective services of prenatal exposure to illegal substances. Knowledge of the consequences of a positive test result creates a dilemma with the performance of reasonable suspicion testing. Under these circumstances testing procedures should include documented chain of custody and automatic confirmation testing of presumptive positives.

### 4.6. What if a donor refutes a positive test result?

Occasionally, a test result is unexpected, does not align with the case, and/or the mother refutes the result. It is a common practice of accredited forensic laboratories to retain positive specimens in an appropriate storage condition (depending on the type of specimen) for an extended period (typically one year) while maintaining chain of custody of the specimen. The purpose of this policy is to allow for the option of retesting the specimen, at the original laboratory or another designated laboratory, to verify the accuracy of the original reported results. This policy provides a safety net of protection for all stakeholders involved.

## 5. Conclusion

Maternal use, misuse, and abuse of substances is a very complicated problem that may initiate lifelong negative consequences for the neonate. Huestis and Choo (56) raised the concern years ago that we need to do more for infants exposed to opioids *in utero* regarding follow-up and appropriate interventions, if needed. It is our responsibility as perinatal healthcare professionals to be aware of the latest developments in the field of toxicology so that we can do the best for the maternal/infant dyad and enrich the lives of those living through the Opioid Epidemic.
